# The association between PaTz and improved palliative care in the primary care setting: a cross-sectional survey

**DOI:** 10.1186/s12875-019-1002-z

**Published:** 2019-08-03

**Authors:** Ian Koper, H. Roeline W. Pasman, Annicka G. M. Van der Plas, Bart P. M. Schweitzer, Bregje D. Onwuteaka-Philipsen

**Affiliations:** Department of Public and Occupational Health, Amsterdam Public Health research institute, Amsterdam UMC, Vrije Universiteit Amsterdam, Van der Boechorststraat 7, 1081BT Amsterdam, The Netherlands

**Keywords:** Health care surveys, Interprofessional relations, Palliative care, Primary health care, Professional-patient relations, Quality of care

## Abstract

**Background:**

The PaTz-method (acronym for Palliatieve Thuiszorg, palliative care at home) is perceived to improve coordination, continuity and communication in palliative care in the Netherlands. Although important for further implementation, research showing a clear effect of PaTz on patient-related outcomes is scarce. This study aimed to examine perceived barriers and added value of PaTz and its association with improved care outcomes.

**Methods:**

Ninety-eight Dutch general practitioners and 229 Dutch district nurses filled out an online questionnaire with structured questions on added value and barrier perception of PaTz-participation, and palliative care provided to their most recently deceased patient, distributed online by Dutch medical and nurses’ associations. Data from PaTz-participants and non-participants was compared using Chi-square tests, independent t-tests and logistic regression analyses.

**Results:**

While both PaTz-participants and non-participants perceived PaTz to be beneficial for knowledge collaboration, coordination and continuity of care, time (or lack thereof) is considered the most important barrier for participation. PaTz-participation is associated with discussing five or more end-of-life topics with patients (OR = 3.16) and with another healthcare provider (OR = 2.55). PaTz-participation is also associated with discussing palliative sedation (OR = 3.85) and euthanasia (OR = 2.97) with another healthcare provider. Significant associations with other care outcomes were not found.

**Conclusions:**

General practitioners and district nurses feel that participating in a PaTz-group has benefits, but perceive various barriers for participation. While participating in a PaTz-group is associated with improved communication between healthcare providers and with patients, the effect on patient outcomes remains unclear. To stimulate further implementation, future research should focus on the effect of PaTz on tangible care characteristics and how to facilitate participation and remove barriers.

## Background

Palliative care, an approach aimed to improve quality of life of patients with a life-threatening illness and their relatives, is complex, focusing on the prevention and relief of suffering from physical, psychosocial, and spiritual issues at the end of life [1]. Contrary to many other western countries like the US, Canada, the UK and Australia, palliative care is not a medical specialty in the Netherlands [2]. It is one of few countries where palliative care is provided in a coordinated care model [3] and national policy states that palliative care should principally be provided by generalists close to patients [4]. In practice, it is often provided by Dutch general practitioners (GPs, in some countries better known as family physicians), who can rely on national guidelines on palliative care provision [5], and on supportive services and facilities such as palliative care consultation teams [6]. However, the ageing population and the increasing numbers of non-acute deaths are likely to lead to a higher demand of palliative care [7], and as GPs are already facing a heavy case-load [8, 9], the provision of good palliative care may be under threat. Furthermore, while multidisciplinary collaboration has shown to be crucial in the delivery of palliative care [10], this is hampered by financial constraints, poor communication and a lack of time [11]. In addition, while communication with patients with a life-threatening disease and relatives on end-of-life topics has consistently been shown to improve quality of care [12–14], GPs struggle to have these conversations with their patients [15].

In recent years, PaTz,^1^ a method aimed to improve palliative care through early identification of palliative patients, early assessment of their needs, symptoms and preferences, and planning care accordingly, has been implemented in the Netherlands. PaTz is an adaption of the British Gold Standards Framework (GSF), a programme aimed at optimising end of life care provision by generalists in all settings including primary care, which has been shown to improve multidisciplinary collaboration, and the consistency and reliability of palliative care in primary care [16]. In local PaTz-groups, GPs and district nurses (DNs, in some countries better known as community nurses) meet bimonthly to identify and discuss their patients with support from a palliative care consultant (a physician or nurse with formal training in palliative care) [17]. A qualitative evaluation study showed that, like the GSF, PaTz is beneficial to healthcare providers: participants felt that it improved cooperation between GPs and DNs, and that it led to better continuity of care, more knowledge on palliative care, and emotional support [17]. A more recent pre-post evaluation study showed again that GPs felt that continuity and coordination of care as well as their own competence to provide palliative care improved after implementation of PaTz [18].

Implementation of PaTz has progressed from four groups at the start in 2012 to more than 160 groups at present. But, as these groups cover only a small part of primary care, further implementation is necessary to improve palliative care in the home setting nationwide. Successful implementation requires understanding of possible barriers for participation, the perceived added value of PaTz, and evidence of the effect on patient related outcomes [19]. However, again like the GSF [20], research showing a clear effect on patient-related outcomes is scarce. The abovementioned pre-post evaluation study also examined the effect of PaTz on aspects of care that are considered important in quality of palliative care: GPs’ awareness of preferred place of death, hospital admission in the final month, treatment goals and GP-patient communication [4, 21–25], but failed to show differences between GPs who did or did not participate in a PaTz group [18]. As some differences were found between patients who were or were not on the PaTz-register or discussed in a PaTz meeting, the authors suggested this might be related to underuse of these important elements of PaTz. Further, they indicated the high level of palliative care before implementation among GPs interested in participating in PaTz might have been influential: a so-called ‘ceiling effect’. The authors recommended therefore including a control group in future studies to be able to account for the latter.

Thus, in order to facilitate further implementation of PaTz, this study first aims to compare PaTz-participants’ perceptions of the added value of PaTz and barriers for participating in PaTz with non-participants’ perceptions. As the roles of GPs and DNs in PaTz-groups differ, and the added value and barriers for participation may be different for GPs and DNs [16, 17], this study aims to compare the perceptions of participants and non-participants separately for each professional group. Second, this study aims to examine the association between PaTz-participation and care outcomes, by comparing the care provided to patients of GPs and DNs participating in a PaTz-group with the care provided to patients of GPs and DNs who are not.

## Methods

### Design

This study is part of a larger project aiming to improve palliative care in the primary care setting. In this project an assessment of the needs and experiences of GPs and DNs with palliative care was performed through an online questionnaire, available online from 5 April 2016 until 5 August 2016. The results of this study were derived from this questionnaire.

### Study population

Potential respondents were invited by professional associations, the national organization of palliative care networks (Fibula) and regional care support networks (ROS) through a call in newsletters and on their websites. Participating professional associations were the Dutch College of General Practitioners (NHG), the Advisory Board of General Practitioners on palliative care (PalHag) and the Dutch Nurses’ Association (V&VN). Respondents were eligible for inclusion if they: 1) were working as a GP or DN in patient care in the Netherlands, 2) had experience with palliative care. In order to assess the representativeness of the sample respondents they were compared to national figures for GPs and DNs on sex, age and working full or part time. The first question participants were asked was whether or not they had cared for a patient with a life-threatening illness or age-related decline in the final phase of their life. If so, they were presented with the rest of the questionnaire. If not, they had no access to the questionnaire.

### Measures

For this study, a questionnaire on perceptions of PaTz, and patient and care characteristics was created in which questions from previous primary palliative care research [18] were used where possible.

### Respondent characteristics

Respondent demographic information included profession, age, gender, employment, years of practice and training in palliative care. After a description of the PaTz-method, respondents were asked whether they participated in PaTz and if not, whether they had heard of the method before.

### Perceptions of added value of PaTz and barriers to participation

Respondents were asked to indicate to what extent they thought PaTz contributes to four aspects of palliative care provision [1]: knowledge [2], coordination [3], continuity and [4] collaboration. Respondents were also asked to indicate to what extent they thought five particular aspects are barriers to participation in PaTz. These aspects were [1]: time [2], financial aspects [3], administration [4], the desire to work alone, and [5] gathering a group of participants. For the purpose of analysis (ensuring sufficient observations in all categories), response options for both questions were: ‘not at all’, ‘hardly’, ‘partly’, ‘greatly’ and ‘don’t know’. The answers were dichotomized by transforming the first two response options into ‘no’, and ‘partly’ and ‘greatly’ into ‘yes’. Missing data was treated as ‘don’t know’. The data from these questions was analysed separately for GPs and DNs.

### Patient and care characteristics

Next, respondents were to report on patient characteristics of their most recently deceased patient, such as age, gender and primary diagnosis and care characteristics, such as hospitalisations in the final two weeks, involved healthcare providers and whether or not the patient died in their preferred place of death. In addition, respondents were asked to report on interdisciplinary communication and communication with the patient, by presenting eight end-of-life topics, for which the respondents were asked to indicate whether they had discussed them with the patient and/or with another healthcare provider. These eight topics were: life expectancy, expected complications, (wishes regarding) hospital admissions, preferred place of death, (wishes regarding) palliative sedation, spiritual issues, treatment options, and (wishes regarding) euthanasia. GPs were asked if they had discussed the topics with a DNs, and vice versa. Again, missing data was treated as unknown.

### Statistical analyses

Chi-square tests and independent sample t-tests were used to compare demographic information between GPs and DNs who participated in PaTz, and GPs and DNs who did not. Chi-square tests were also used to compare perceptions on the added value of and barriers for participation in PaTz between these two groups. Logistic regression analyses were used to compare the characteristics of patients described by either group, as well as the topics discussed and other care characteristics. First, crude logistic regression analysis were performed with being a PaTz participant or not as independent variable and the difference care characteristics as dependent variable. In order to adjust for healthcare provider characteristics that differed between health care providers who did and did not participate in PaTz, we performed multivariable analyses in which these characteristics were added as independent variables. We present the results of these analyses as Odds Ratios with respective 95%-confidence intervals. All statistical analyses were performed using SPSS, IBM Statistics for Windows version 22.

## Results

### Sample characteristics

The characteristics of the 327 respondents are shown in Table 1. The majority was female (86%), and their mean age was 47 years. Most worked part-time (77%), averaging 26 h per week. The mean years in practice was 14 (13 for DNs and 17 for GPs), and 58% of the respondents had received training in palliative care. GPs participated in a PaTz-group more often than DNs (70% vs 28%), while 23 and 35% of the non-participating GPs and DNs had not heard of PaTz before. When comparing the characteristics between PaTz-participants and non-participants, the only significant difference was found in DNs’ employment: DNs not participating in PaTz more often worked part-time. Nationwide, the mean age of GPs is 48 years, and 51% are women [26], while the mean age of DNs is 45 years, and 92% are women [27]. Thus, compared with these national figures, our sample was of similar age while consisting of a high proportion of female GPs. While all respondents reported on the barriers and added value of PaTz, 98 were unwilling to report on their most recently deceased patient.

**Table 1:** Characteristics of 327 Dutch respondents in the online questionnaire on PaTz-participation and palliative care

Characteristics^a^	Total	General practitioners (*n* = 98)		District nurses (*n* = 229)	
	*N* = 327	PaTz *N* = 69 (70%)	No PaTz *N* = 29 (30%)	PaTz *N* = 64 (28%)	No PaTz *N* = 165 (72%)
Female gender, N (%)	280 (86%)	50 (73%)	17 (59%)	57 (91%)	156 (95%)
Age, (mean (SD))	47 (10.6)	50 (8.5)	47 (8.9)	46 (11.3)	46 (11.1)
Working part-time, N (%)	252 (77%)	51 (74%)	18 (62%)	37 (58%)^1^	146 (89%)^1^
Part-time hours per week, mean (SD)	26 (8.6)	31 (8.7)	30 (5.6)	24 (8.3)	24 (8.2)
Working experience, mean years (SD)	14 (10.4)	18 (8.9)	15 (10.0)	13 (8.9)	13 (11.1)
Training in palliative care, N (%)	190 (58%)	37 (54%)	14 (48%)	39 (61%)	100 (61%)

^a^Missing data < 5% for each variable

^1^Statistically significant difference found for ‘working part-time’ in DNs between PaTz and No PaTz (*p* < 0.001)

### Perceptions of added value of PaTz and barriers to participation

Table 2 provides GP and DN perceptions of added value of PaTz and barriers to participation in PaTz. The percentage of GPs and DNs perceiving added value of PaTz was relatively high for all four aspects of palliative care, ranging from an average of 85% for ‘continuity’ to 96% for ‘knowledge’ and ‘collaboration’. Except for ‘collaboration’, no statistically significant differences between perceptions of added value of PaTz were found between GPs and DNs participating in PaTz and those who were not. Overall, ‘time’ was most often considered a barrier for participation (84%), whereas ‘desire to work alone’ was least often perceived to hinder participation (16%). When comparing perceptions of barriers between GPs, we found that non-participants more often perceived ‘time’ (100% vs 88%) and ‘administration’ (73% vs 50%) as a barrier for participation than their participating colleagues. For ‘financial aspects’, ‘the desire to work alone’ and ‘finding a group’ we found no statistically significant difference. When comparing perceptions of barriers for participation between DNs, we found that non-participating DNs perceived all aspects as barrier for participation more often than DNs who were.

**Table 2:** Perceived added value of PaTz and perceived barriers for participation of 98 GPs and 229 DNs in the Netherlands

Aspect	Total *N* = 327 (yes (%))	General practitioners		District nurses	
		PaTz *N* = 69 (yes (%))	No PaTz *N* = 29 (yes (%))	PaTz *N* = 64 (yes (%))	No PaTz *N* = 165 (yes (%))
PaTz is of added value to^a^					
Knowledge	310 (96)	63 (93)	28 (97)	62 (98)	157 (96)
Collaboration	303 (96)	66 (99)^1^	26 (90)^1^	63 (98)	148 (95)
Coordination	279 (88)	55 (83)	24 (83)	60 (94)	140 (89)
Continuity	270 (85)	51 (76)	18 (64)	57 (92)	144 (90)
Barrier for participation^b^					
Time	258 (84)	59 (89)	29 (100)	38 (62)^2^	133 (87)^2^
Finding a group	179 (66)	31 (53)	19 (70)	28 (50)^2^	101 (78)^2^
Financial aspects	166 (62)	33 (51)	17 (68)	28 (50)^3^	88 (72)^3^
Administration	163 (60)	32 (50)	17 (71)	21 (36)^2^	93 (72)^2^
Desire to work alone	42 (16)	7 (11)	2 (8)	5 (9)^4^	28 (22)^4^

^a^Missing data < 5% for all added values variables

^b^Missing data for ‘time’ < 1%, for other barrier variables between 13 and 14%

^1^Statistically significant difference found for ‘collaboration’ in GPs (*p* = 0.046)

^2^Statistically significant difference found for ‘time’, ‘finding a group’, and ‘administration’ in DNs (*p* < 0.001)

^3^Statistically significant difference found for ‘financial aspects in DNs (*p* = 0.004)

^4^Statistically significant difference found for ‘desire to work alone’ (*p* = 0.044)

### Characteristics of patients described by PaTz-participants and non-participants

The characteristics of the described patients are shown in Table 3. The mean age at death was 70–72 years, and 53–55% was female. Most patients were diagnosed with cancer (62–70%), and the majority had been living at home (89–90%). We found no statistically significant differences between the patients described by GPs and DNs participating in PaTz and those who were not in the crude nor in the adjusted analysis.

**Table 3:** Characteristics of patients described by 93 PaTz-participants and 142 non-PaTz-participants in the Netherlands

Characteristics^a^	Total (*n* = 235)	PaTz (*n* = 93)	No PaTz (*n* = 142)	Crude OR^b^ (95% CI)	Adjusted OR^c^ (95%CI)
Age at death (years)					
65 or younger (ref)	71 (31%)	28 (31%)	43 (31%)	1	1
66–75	60 (26%)	29 (32%)	31 (22%)	1.44 (0.72–2.88)	1.12 (0.51–2.43)
76–85	60 (26%)	20 (22%)	40 (29%)	0.77 (0.38–1.57)	0.69 (0.31–1.53)
86 or older	40 (17%)	14 (15%)	26 (19%)	0.83 (0.37–1.85)	0.73 (0.29–1.85)
Gender (% female)	55%	53	55	0.92 (0.54–1.6)	0.76 (0.42–1.4)
Diagnosis					
Cancer (ref)	153 (65%)	65 (70%)	88 (62%)	1	1
Cardiovascular disease	4 (2%)	2 (2%)	2 (1%)	1.4 (0.19–9.9)	0.83 (0.07–10.3)
COPD	8 (3%)	3 (3%)	5 (4%)	0.81 (0.19–3.5)	1.3 (0.26–6.0)
Stroke	3 (1%)	0	3 (2%)	0	0
Dementia	2 (1%)	1 (1%)	1 (1%)	1.4 (0.08–22.1)	1.3 (0.06–30.5)
Frailty/age-related decline	10 (4%)	3 (3%)	7 (5%)	0.58 (0.14–2.3)	1.2 (0.27–5.3)
Multi-morbidity	53 (23%)	18 (19%)	35 (25%)	0.70 (0.36–1.3)	0.81 (0.39–1.7)
Other	2 (1%)	1 (1%)	1 (1%)	1.4 (0.08–22.1)	3.5 (0.21–58.3)
Setting					
Home (ref)	191 (90%)	75 (89%)	116 (90%)	1	1
Residential care home	12 (6%)	3 (4%)	9 (7%)	0.52 (0.14–2.0)	0.27 (0.06–1.2)
Hospice	10 (5%)	6 (7%)	4 (3%)	2.3 (0.63–8.5)	2.0 (0.48–8.4)

^a^Missing data < 2% for each variable

^b^OR = Odds ratio

^c^Adjusted for healthcare providers’ profession (GP or DN) and employment (part-time or full-time)

### Characteristics of care provided by PaTz-participants and non-participants

Table 4 provides an overview of the topics discussed and care characteristics of the patients as described by the respondents and their relationship to PaTz-participation. Logistic regression analyses were adjusted for healthcare providers’ profession (GP or DN) and employment (part-time or full-time). While some GPs and DNs had discussed 0–1 (21–24%) and 2–4 of the topics (19–28%) with another healthcare provider, a substantial part (37–44%) had discussed 5 or more of the presented topics with another healthcare provider. Logistic regression analysis showed that PaTz-participation was significantly associated with discussing 5 or more topics (OR = 2.55, 95% CI = 1.11–5.88). The same pattern applies to PaTz-participation and the number of topics discussed with the patient. While few GPs and DNs (5–16%) had discussed 0–1 of the topics, and a minority (11–15%) had discussed 2–4 topics, most GPs and DNs (69–84%) had discussed 5 or more topics with the patient. Again, logistic regression analysis showed a significant association between PaTz-participation and discussing 5 or more topics (OR = 3.16, 95% CI = 1.04–9.64). Regarding the relationship between PaTz-participation and the discussion of specific topics, logistic regression analysis showed that PaTz-participation was significantly associated with discussing ‘(wishes regarding) palliative sedation’ (OR = 3.85, 95% CI = 1.71–8.66) and ‘(wishes regarding) euthanasia’ (OR = 2.97, 95% CI = 1.48–5.97) with another healthcare provider. The significant associations between PaTz-participation and topics discussed with patients found in the crude analysis disappeared in the adjusted analysis, indicating that PaTz-participation was not associated with the discussion of particular topics with patients.

**Table 4:** Results from logistic regression analyses estimating the association between PaTz-participation and characteristics of palliative care provided to their most recently deceased patient by 235 Dutch healthcare providers

Care characteristics^a^	Total (*n* = 235)	PaTz (*n* = 93)	No PaTz (*n* = 142)	Crude OR (95% CI)	Adjusted OR^b^ (95% CI)
Number of topics discussed with another healthcare provider					
0–1 topics	63 (27%)	26 (28%)	37 (26%)	1	1
2–4 topics	78 (33%)	26 (28%)	52 (37%)	0.71 (0.26–1.42)	1.31 (0.57–2.99)
5–8 topics	94 (40%)	41 (44%)	53 (37%)	1.10 (0.58–2.10)	2.55 (1.11–5.88)*
Topics discussed with another healthcare provider					
Life expectancy	150 (64%)	60 (65%)	90 (63%)	1.05 (0.61–1.81)	1.75 (0.91–3.37)
Expected complications	137 (58%)	57 (61%)	80 (56%)	1.23 (0.72–2.09)	1.41 (0.75–2.63)
(Wishes regarding) palliative sedation	137 (58%)	57 (61%)	80 (56%)	1.23 (0.72–2.09)	3.85 (1.71–8.66)**
(Wishes regarding) hospital admission	113 (48%)	46 (50%)	67 (47%)	1.10 (0.65–1.85)	1.60 (0.86–2.98)
Treatment options	107 (46%)	40 (43%)	67 (47%)	0.85 (0.50–1.43)	1.24 (0.66–2.30)
Preferred place of death	102 (43%)	43 (46%)	59 (42%)	1.21 (0.71–2.05)	1.70 (0.91–3.17)
(Wishes regarding) euthanasia	82 (35%)	37 (40%)	45 (32%)	1.42 (0.83–2.46)	2.97 (1.48–5.97)**
Spiritual issues	45 (19%)	17 (18%)	28 (20%)	0.91 (0.47–1.78)	1.41 (0.66–3.01)
Number of topics discussed with patient					
0–1 topics	28 (12%)	5 (5%)	23 (16%)	1	1
2–4 topics	31 (13%)	10 (11%)	21 (15%)	2.19 (0.64–7.46)	2.95 (0.77–11.3)
5–8 topics	176 (75%)	78 (84%)	98 (69%)	3.66 (1.33–10.1)*	3.16 (1.04–9.64)*
Topics discussed with patient					
Life expectancy	191 (81%)	79 (85%)	112 (79%)	1.51 (0.75–3.03)	1.21 (0.56–2.63)
Expected complications	161 (69%)	71 (76%)	90 (63%)	**1.87** (**1.04–3.36**)*	1.60 (0.84–3.07)
(Wishes regarding) hospital admission	185 (79%)	82 (88%)	103 (73%)	**2.82** (**1.36–5.85**)**	1.68 (0.75–3.74)
Preferred place of death	185 (79%)	80 (86%)	105 (74%)	**2.17** (**1.08–4.35**)*	2.05 (0.95–4.41)
(Wishes regarding) palliative sedation	168 (72%)	75 (81%)	93 (66%)	**2.20** (**1.18–4.08**)*	1.70 (0.86–3.36)
Spiritual issues	155 (66%)	62 (67%)	93 (66%)	1.05 (0.61–1.83)	1.17 (0.63–2.18)
Treatment options	164 (70%)	72 (77%)	92 (65%)	**1.86** (**1.03–3.38**)*	1.24 (0.64–2.43)
(Wishes regarding) euthanasia	144 (61%)	68 (73%)	76 (54%)	**2.36** (**1.34–4.16**)**	1.56 (0.83–2.94)
Healthcare provider expecting the patient’s death					
More than 6 months in advance (ref)	52 (22%)	23 (25%)	29 (21%)	1	1
3–6 months in advance	68 (29%)	29 (31%)	39 (28%)	0.94 (0.45–1.94)	1.19 (0.51–2.74)
1–2 months in advance	41 (18%)	14 (15%)	27 (19%)	0.65 (0.28–1.52)	0.83 (0.31–2.17)
In the final month	45 (19%)	17 (18%)	28 (20%)	0.77 (0.34–1.73)	1.63 (0.64–4.13)
In the final week	27 (12%)	10 (11%)	17 (12%)	0.74 (0.29–1.93)	1.53 (0.52–4.51)
Hospital admission in the final 2 weeks	45 (19%)	20 (22%)	25 (18%)	1.26 (0.65–2.43)	1.60 (0.77–3.35)
Preferred place of death known	226 (96%)	89 (96%)	137 (96%)	1.30 (0.23–7.24)	0.95 (0.15–5.93)
Died at preferred place of death	219 (93%)	85 (91%)	134 (94%)	0.48 (0.10–2.18)	0.76 (0.15–3.95)
Place of death					
Home (ref)	175 (75%)	72 (78%)	103 (74%)	1	1
Residential care home	18 (8%)	5 (5%)	13 (9%)	0.55 (0.19–1.61)	0.59 (0.18–1.93)
Hospice	27 (12%)	10 (11%)	17 (12%)	0.84 (0.36–1.94)	0.76 (0.30–1.93)
Hospital	8 (3%)	4 (4%)	4 (3%)	1.43 (0.35–5.91)	0.88 (0.18–4.26)
Other/don’t know	4 (2%)	1 (1%)	3 (2%)	0.48 (0.05–4.67)	0.33 (0.03–3.95)

^a^Missing data < 2% for all variables

^b^Adjusted for healthcare providers’ profession (GP or DN) and employment (part-time or full-time)

* *p* <0.05 ** *p* <0.01

Considering the other care characteristics, no significant differences were found between PaTz-participants and non-participants. Around half (49–56%) of the GPs and DNs expected the patients’ death 3 months in advance or earlier while a minority (11–12%) did not expect the patient’s death until the final week. Further, almost all GPs and DNs (97–98%) were aware of their patient’s preferred place of death and the vast majority of patients (93–95%) died at their preferred place. Most patients (74–78%) died at home, and even though one fifth (18–22%) patients was admitted to a hospital in the final 2 weeks, only a small minority (3–4%) died there. Logistic regression analysis showed no significant association between PaTz-participation and any of these care characteristics.

## Discussion

### Summary of findings

Respondents considered PaTz to be of value on all four prompted aspects: knowledge, coordination, continuity of care and collaboration. A lack of time was considered the most important barrier for participation in PaTz, but financial aspects, administrative burden and having to find a group to participate in were also perceived as barriers by the majority of respondents. While we found an association between participating in a PaTz-group and discussing more topics with another healthcare provider and with patients, we found no associations between PaTz-participation and other care characteristics.

### Strengths and limitations of this study

A strength of this study is that, contrary to prior evaluation studies, a control group was included. Still, even though the cross-sectional design of this study can demonstrate associations between PaTz and care outcomes, it is not suitable to demonstrate causality. Another limitation of this study lies in the recruitment strategy. While our recruitment strategy enabled GPs and DNs from all over the country to participate, it does not allow for response rates to be calculated. Also, as the care characteristics were self-reported by GPs and DNs, recall bias could play a role and the patient perspective is underexposed. Finally, it is possible GPs and DNs interested in palliative care are overrepresented in the sample. This could lead to an overly positive image of palliative care in the primary care setting, and an underestimation of the effect of PaTz.

### Comparison and reflection

A large majority of both participants and non-participants recognized the benefits of PaTz regarding knowledge, coordination, continuity and collaboration. PaTz-participants, particularly nurses, generally saw fewer barriers for participation, but regardless of participation or profession, it is clear that a lack of time is the most important one. It is possible that participating in a PaTz-group leads to reduced barrier perception, but it could also be the other way around: participation *requires* reduced barrier perception. However it may be, the reduced barrier participation and unchanging added value perception of PaTz-participants can be used in the promotion of PaTz. Still, further in-depth qualitative exploration of the benefits of PaTz, how to increase value and how to remove barriers for participation is recommended.

Further, the results showed that, like participating in the GSF [28], PaTz-participation seems to be associated with improved communication with other healthcare providers as PaTz-participants more often discussed 5 or more topics relevant to palliative care than non-participants. Similarly, as PaTz-participants more often discussed 5 or more topics with their patient, PaTz-participation seems to be associated with improved communication with the patients. As end-of-life communication between GPs and DNs and with patients is crucial to the delivery of adequate palliative care [12, 13, 29, 30], these are important findings.

Still, beside the number of topics discussed, we found no differences in care characteristics. A ceiling effect, as suggested by Van der Plas [18], could be the cause, as the level of palliative care provided was generally high. Over two thirds of the respondents expected the patients’ death more than a month in advance, providing time to plan and deliver effective end-of-life care [31]. Also, nearly all respondents were aware of the preferred place of death of the patient (96%), even though the percentage of GPs and DNs that reported to have discussed the topic with the patient was somewhat lower at 74–86%. Finally, most patients died at their preferred place (93%). Despite being self-reported and the possibility of recall bias, these numbers are impressive compared to earlier studies, where the patients preferred place of death was known in 54–60% of the cases and approximately 80% died at their preferred place [32, 33]. This high level of palliative care may not be representative for the general level of palliative care in primary care, and healthcare providers with less affinity for palliative care may benefit more from PaTz. It should also be mentioned that, for reasons unknown to us, 30% of the respondents did not report on their most recent case. Next to merely not wanting to spent more time on the questionnaire it is also possible that for some their most recent case concerned patients where the care was managed less than ideal. Further, it is possible that respondents who did provide patient and care details, reported on a recent patient whose care was managed well, rather than their actual most recent case.

Overall, while this study has shown a few promising associations, we recommend future research to focus on the effect of PaTz on tangible care outcomes in a design suitable to show causality, and on the perspective of patients and relatives on the care provided and how to facilitate participation and remove barriers in a qualitative manner.

## Conclusions and practical implications

Confirming the previously reported perception of participants that PaTz improves communication in palliative care [17, 18], this article adds to the body of evidence of the value of PaTz in the primary care setting. As communication with other healthcare providers and with patients is key in palliative care [12, 34], participating in PaTz can aid healthcare providers in their task. Tailored to country-specific health care systems, this may also be in the case in other countries where generalists are the primary palliative care providers, like Canada, Australia, Belgium, Italy and Spain [35–37].

Our study also shows that further implementation of PaTz is barred by GPs’ and DNs’ perceived lack of time, and financial compensation and involves additional administrative red tape. Targeted promotion of PaTz by colleagues sharing success stories and positive experiences and firm evidence of its effects, could facilitate adoption of the method. In addition, as PaTz is more likely to benefit healthcare providers with less affinity with palliative care, implementation of PaTz in that particular group deserves extra attention.

## Data Availability

The dataset used and analysed during the current study are available from the corresponding author on reasonable request.

## Abbreviations

DN: District nurse
GP: General practitioner
GSF: Gold Standards Framework
OR: Odds Ratio
PaTz: PAlliatieve ThuisZorg (palliative care at home)

## Footnotes

1 PaTz is an acronym for PAlliatieve ThuisZorg (palliative care at home)
